# Research on the Mechanism and Prevention Methods of the Drying Shrinkage Effect of Earthen Sites

**DOI:** 10.3390/ma15072595

**Published:** 2022-04-01

**Authors:** Zehuan Zhang, Jianzhong Yang, Jianwei Yue, Wenhao Li, Huijie Gao

**Affiliations:** 1 School of Civil Engineering, Zhengzhou University, Zhengzhou 450001, China; zehuanzhangtom@163.com; 2 School of Civil Engineering and Architecture, Henan University, Kaifeng 475004, China; yjwchn@126.com (J.Y.); l308727900@126.com (W.L.); g18533929521@163.com (H.G.)

**Keywords:** expansive soil, expansibility and contractility, crack, wet and dry cycle, lime improvement, waterproof

## Abstract

In view of the fact that it is easy for the ancient city soil site of Cai Kingdom to expand and crack when encountering water, this paper explores the methods to improve the expansion and shrinkage deformation, dry shrinkage cracks and easy water absorption characteristics of the expanded site soil based on a lime and silicone hydrophobic agent. In this paper, the expansive clay in the old city site of Cai Kingdom in Zhumadian was taken as the research object, and the dry-shrinkage fissure test of saturated expansive soil was carried out, to study the influencing factors of the dry-shrinkage cracking of expansive soil in this area. The site soil was modified with lime and glue powder, and the fissure image was quantitatively analyzed by MATLAB. The test shows that the smaller the particle size, the faster the evaporation of water and the smaller the surface fissure rate; the thicker the thickness of the soil sample, the greater the surface fissure rate and the greater the crack width; and with the increase in the number of drying and wetting cycles, the surface fissure rate of the soil sample increases. In this paper, lime and waterproof materials are used to improve the expansive soil. This not only reduces the dry shrinkage crack rate, but also improves the waterproof performance and durability of expansive soil.

## 1. Introduction

Earthen sites are an important part of China’s cultural relics and an important basis for studying China’s historical culture. In Henan Province, there are many important earthen sites in many areas, and the soil in the area is geological. Expansive soils, also known as expansion and contraction soils, have the characteristics of volume expansion following water absorption and volume contraction following water loss. Expansive soil contains more mineral components, such as montmorillonite and Illite, so it is extremely hydrophilic. In the dry season, the soil loses water and shrinks, resulting in cracks [1,2], and cracks caused by soil shrinkage reduce the carrying capacity and overall stability of the soil, increase the compressibility of the soil [3], and dramatically increase the permeability of the soil [4,5]. The soil sites are mostly open air immovable cultural relics, which have been subjected to wind erosion, rain erosion, and alternating dry and wet conditions for a long time, resulting in cracks on the surface of the site soil, reduced mechanical properties, the reduced stability of cultural relics, and unsightly surfaces [6]. The earth site was buried deep underground before being excavated and has a high water content. As the excavation process progresses, soil cultural relics are gradually exposed to dry air, and, subsequently, water loss, shrinkage, cracking, and cracks occur, destroying the integrity of the cultural relics [7,8].

At present, the research on expansive soils is mainly divided into three directions: (1) theoretical model research on the expansion and contraction of expansive soil; (2) experimental studies on the expansion and contraction of expansive soil, and analysis of the factors affecting the expansion and contraction of expansive soil; and (3) improvement for the expansion and contraction characteristics of expansive soils. Based on the existing VSC model, Dai et al. [9] proposed a model for describing the shrinkage deformation fit of the expansive soil under different initial states, and combined three different shrinkage tests to verify the model, meaning the fitting results were more consistent with the experimental data. Wei et al. [10] believed that the volume change in compacted expansive soil under the action of drying and wetting cycles can be divided into a reversible component and an irreversible component, combined with the BBM model, a constitutive model suitable for solidifying expansive soil was proposed. Huang et al. [11] took Nanyang expansive soil as the research object, and realized the triaxial stress state expansion test by modifying the test device. On this basis, a triaxial stress state expansion model was established, and the model was applied to the numerical calculation. The verification shows that the model expression is concise, the parameter calculation is simple, and the practicability is strong. Li et al. [12] took Hefei black medium expansive soil as the research object. After the dry–wet cycle test was carried out on the soil sample, the crack morphology was quantitatively analyzed, and it was concluded that the damage of the dry–wet cycle to the soil sample was concentrated in the first two drying processes. The increase in the number of wet cycles has no obvious damage to the surface fissures, and is mainly used to develop the fissure depth. Tang et al. [13] studied the shrinkage cracking characteristics of expansive soil under different temperature conditions, and carried out a series of drying experiments, which showed that the evaporation rate of water in soil samples increased with the increase in temperature, and the moisture content is significantly affected by temperature when the cracks appeared. Yuan et al. [14] carried out a series of comparative experiments on expansive soil, lime modified expansive soil, and steel slag improved expansive soil, and concluded that steel slag had obvious improvement effects on the development of cracks, the reduction in free expansion rate and the increase in the strength of the expansive soil. Fu et al. [15] used fiber fly ash to improve expansive soil, and conducted an unconfined compressive strength test. The test results show that the strength of the expansive soil modified by fiber fly ash is improved, and with the increase in the curing age, the strength of the modified soil increases gradually.

The formation and development of fissures is a major factor in the destruction of soil structure [16,17]. The above mentioned existing research on fissures and improvements in expansive soil only focuses on improving strength and mechanical properties, ignoring improving the waterproofness of soil. The existing research focuses on the impact of dry–wet cycles on the development of fissures, while ignoring the impact of the particle size and thickness of soil on fissure formation. This paper takes the expansive site soil of Cai Guo Ancient City in Zhumadian as the object, and conducts experiments from the two starting points of improving shrinkage cracks and improving the waterproofness of the site soil, in order to study the effects of soil particle size, soil layer thickness and the dry–wet cycle on dry shrinkage cracks.

## 2. Soil Sample Preparation and Test Methods

**Figure 1:**
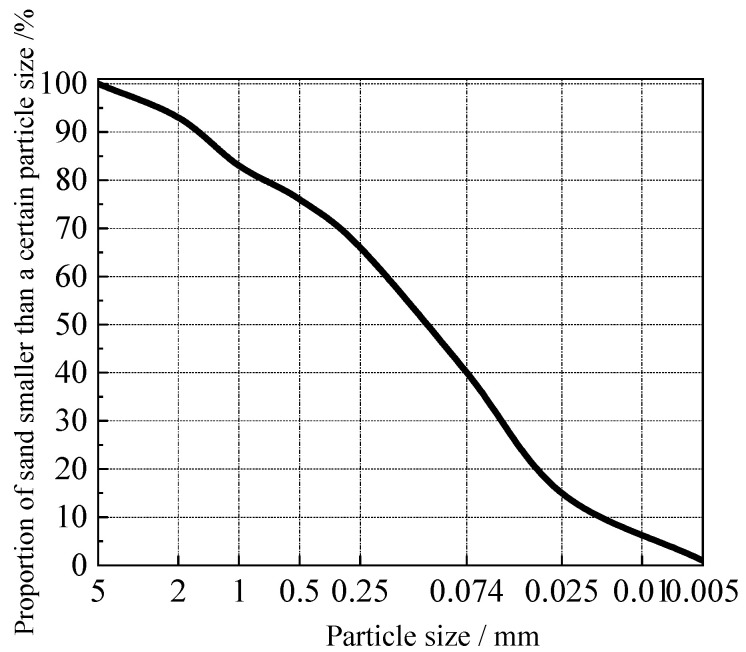
Grain gradation test results.

The swelling soil used in this test was taken from the soil site of the former city of Caiguo in Zhumadian. The Cai Guo Forbidden City was built in the Western Zhou period, with a rectangular shape and a perimeter of 10409 m. The soil is a swelling soil with a strong hydrophilic nature, due to the local material, and it swells with water absorption and shrinks with water loss. Geotechnical tests were carried out on the retrieved site soil in accordance with the Standard for Geotechnical Test Methods (GB/T50123-2019). The basic physical indicators of the soil samples are shown in Table 1, and the sieving results of the soil samples are shown in Figure 1. The dry shrinkage fissure test was carried out on the saturated soil to study the fissure development of the site soil under different soil sample particle sizes, soil layer thickness and wet and dry cyclic conditions, and to compare with and analyse the site soil amended with lime and silicone water repellents.

### 2.1. Soil Sample Preparation

(1) Sample preparation process for the plain soil group

① The retrieved site soil was air dried, crushed and passed through 1mm and 5mm sieves, respectively, for reserving [18]. ② The sieved soil samples were then placed in a 30cm diameter circular mould and water was added to prepare a saturated slurry sample with a water content of 59.1% (1.5 times the liquid limit [19]). ③ The sample was mixed well and shook on a shaker for 5 min and vacuumed through a vacuum saturation cylinder to remove air bubbles from the interior of the slurry. ④ It was covered with cling film and sealed in a container and left to stand for more than 12 h, with the aim of allowing the mineral components of the soil sample to fully absorb water.

All test specimens were tested in circular moulds with a diameter of 30 cm. When conducting tests with different soil thicknesses, the thickness of the soil layer was 3 cm for specimens in groups S7–S9 and 1 cm for all other test groups.

(2) Sample preparation process for improved groups

This test uses modified materials: lime and silicone hydrophobic agents. Lime dissolved in water produces a colloid and reacts with carbon dioxide in the air to produce calcium carbonate crystals to improve the strength of the soil sample to reduce dry shrinkage cracks; the silicone hydrophobic agent is mainly composed of silane and dendrimer and is a good water repellent material to improve the water resistance of the soil sample. The sample preparation process for the plain soil group was repeated, and the modified materials were added to the dry soil at the same time in a certain proportion (all at 5% of the dry soil mass), and water was added to prepare a modified saturated slurry sample with a water content of 59.1% (1.5 times the liquid limit [18]).

(3) Dry and wet cycle test

① The dry and wet cycle specimen preparation process was the same as that for the plain soil group. For the wet and dry cycle tests, the initial moisture content was 59.1% (1.5 times the liquid limit [19]) and the sample was placed in an oven for drying until the specimen mass remained constant and the first wet and dry cycle process was completed. ② During the second and third wet and dry cycles, water was added to the specimen again by calculating the water content lost from a water content of 59.1% (1.5 times the liquid limit [19]) and placed in the oven for drying until the specimen mass remained the same and the wet and dry cycle test was completed.

### 2.2. Soil Sample Preparation

The improved materials used in the test are calcium oxide powder and American Dow Corning waterproof emulsion. The two improved materials are nontoxic, and will adhere to the surface of soil materials and pore surfaces, which are not easily washed away. Lime calcium powder is limestone with calcium carbonate as the main component, which is calcined at high temperature to become quicklime, and then partially digested. The main components are calcium hydroxide and calcium oxide, as well as a small amount of calcium carbonate. The Dow Corning waterproof emulsion is an organosilicon hydrophobic agent. The silicone hydrophobic coating is made of silane and siloxane as the main raw material. It is a new type of highly effective waterproof material that is penetrating, nontoxic, pollution free and nonirritating.

The methyl group in sodium methylsilicate molecules is a common hydrophobic group, which can form an excellent hydrophobic layer on the surface of soil particles, and has the function of micro-expansion and increasing the compactness of soil. Sodium methylsilicate reacts with water and CO_2_ to form polysiloxane film, which has strong hydrophobicity, and can increase the contact angle of soil particle surface and improve its water resistance. Its chemical reaction formula is as follows.

(1) $${2\mathrm{CH}}_{3}{\mathrm{Si}(\mathrm{OH})}_{2}{\mathrm{ONa}+\mathrm{CO}}_{2}{+H}_{2}O\to {2[\mathrm{CH}}_{3}{\mathrm{Si}(\mathrm{OH})}_{3}{]+\mathrm{Na}}_{2}{\mathrm{CO}}_{3}$$

(2) $${n[\mathrm{CH}}_{3}{\mathrm{Si}(\mathrm{OH}}_{3})]\to {{[\mathrm{CH}}_{3}{\mathrm{SiO}}_{\frac{3}{2}}]}_{n}+\frac{3}{2}H_{2}O$$

Lime can crystallize and carbonize in soil to form Ca(OH)_2_ crystals and CaCO_3_ precipitation. Ca(OH)_2_ crystal is a solid with high strength, which can improve the mechanical properties of the sample. A part of CaCO_3_ can be cemented with soil particles, increase the compactness of soil samples, reduce its porosity and improve the binding force between soil particles.

### 2.3. Effect of Particle Size on Dry Shrinkage Fissures

In this paper, when studying the influence of particle size on the crack development and morphology of expansive site soil, S1–S3 are soil samples passing 1 mm sieve, and S4–S6 are soil samples passing 5 mm sieve. We took out the soil samples at regular intervals for quality measurement, took photos of the soil samples from the same fixed height, and recorded the crack development.

**Figure 2:**
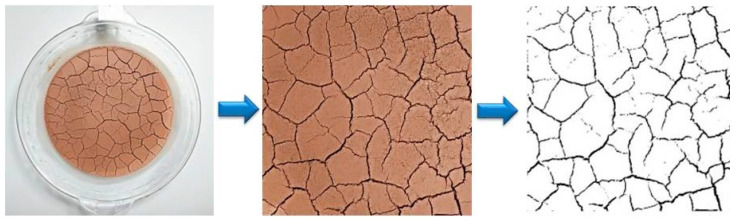
Image processing process.

For better fissure development comparison, and subsequent image processing, it is necessary to process the pictures recorded during the test; for the pictures of each soil sample, only the pictures with a fixed pixel size of the central soil sample were retained. In this paper, by using Image Pro Plus 6.0 software to intercept images taken during the drying process, and then using the image processing function in Matlab software to binarise and noise reduce the intercepted images, we ended up with images with only black and white pixels, with black pixels representing cracks and white pixels representing clods of soil (Figure 2). A program was written to calculate the ratio of the area of the black pixels to the total pixels, which is the fracture rate. The fracture rate is used as a metric to quantify the fracture network and is used to analyse the effect of the factors on the fracture network in this experimental design.

**Figure 3:**
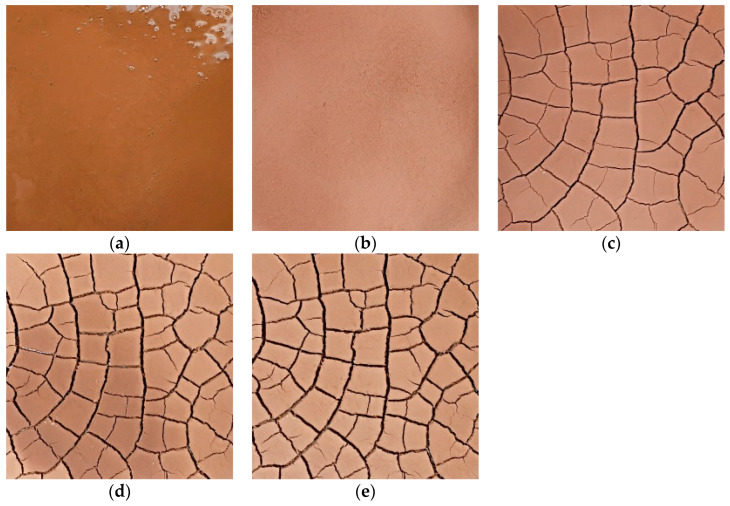
Fracture development morphology of soil sample of group S1 specimens. (**a**) 0 h; (**b**) 1 h; (**c**) 2 h; (**d**) 2.5 h; (**e**) 2.6 h.

**Figure 4:**
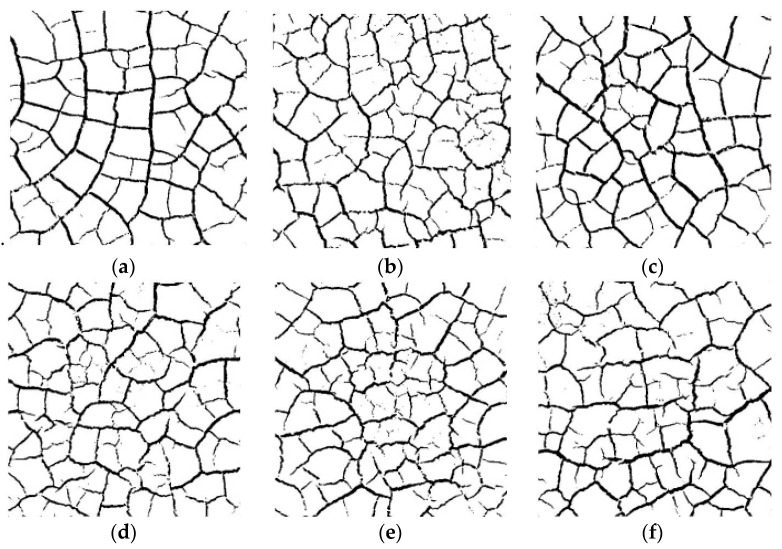
Developmental morphology of two particle size dry shrinkage fissures. (**a**) Group S1 specimens; (**b**) Group S2 specimens; (**c**) Group S3 specimens; (**d**) Group S4 specimens; (**e**) Group S5 specimens; (**f**) Group S6 specimens.

Without compromising the quality of the reader’s reading, the pictures of the cracking process of the S1 group of specimens are listed separately as examples for all test groups (Figure 3), while the pictures of the other groups are shown in the text only for the final cracking. Figure 4 shows the development process of dry shrinkage fissures with particle size of 1 mm and 5 mm. As can be seen from Figure 4, the soil sample loses water and shrinks at a certain temperature, resulting in some obvious main cracks and some small secondary cracks, which destroy the integrity of the soil sample surface and the stability of the structure.

### 2.4. Effect of Dry and Wet Cycle on Dry Shrinkage Fissures

**Figure 5:**
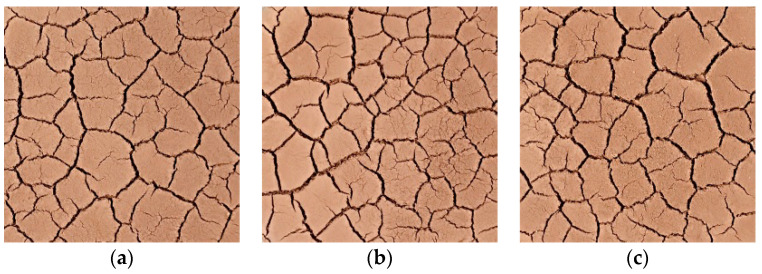
Fracture development morphology of soil sample in the second dry–wet cycle. (**a**) Group S1 specimens; (**b**) Group S2 specimens; (**c**) Group S3 specimens.

**Figure 6:**
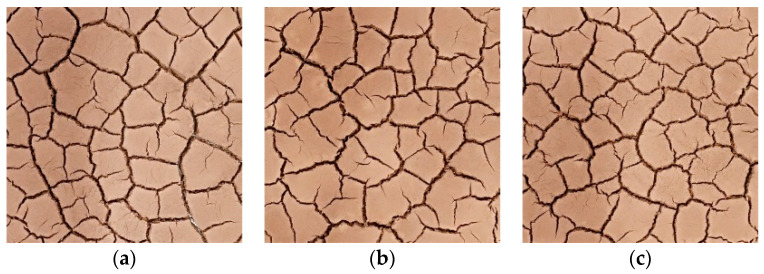
Fracture development morphology of soil sample after three dry–wet cycles. (**a**) Group S4 specimens; (**b**) Group S5 specimens; (**c**) Group S6 specimens.

To explore the effect of dry and wet cycles on the dry shrinkage fissures of soil samples, the dry and wet cycles were carried out on dry shrinkage soil samples S1–S3. Figure 5 and Figure 6 show the fissure morphology after second and third dry and wet cycles of S1–S3, respectively. It can be seen from Figure 5 and Figure 6 that, comparing the pictures of the three wet–dry cycles, it is found that, with the increase in the number of dry–wet cycles, the fissures continue to develop and expand, the width of the main fissure increases, and the secondary fissures produced by the soil samples under the action of dry–wet cycles also gradually develop.

### 2.5. Effect of Soil Sample Thickness on Dry Shrinkage Fissure

**Figure 7:**
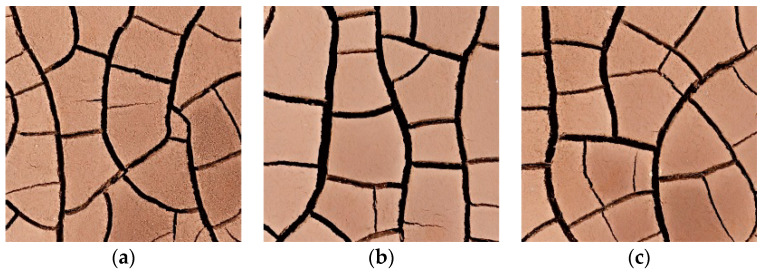
Soil-like fissure development morphology. (**a**) Group S7 specimens; (**b**) Group S8 specimens; (**c**) Group S9 specimens.

The objective of different soil sample thicknesses is achieved by controlling the weight of the soil samples used in the test. Figure 7 shows the crack development morphology of S7–S9 soil samples with a soil layer thickness of 3 cm. Compared with the fissure morphology of soil samples S1–S3 in Figure 4, it can be clearly seen that the width of dry shrinkage crack produced by soil sample with soil layer thickness of 3 cm is significantly wider, and there are only a few tiny secondary fissures. Intuitive observation shows that the thicker the thickness, the wider the shrinkage fissures will be, and the fissures will penetrate the surface of the soil sample and connect with each other.

### 2.6. Dry Shrinkage Fissure Test of Improved Soil

**Figure 8:**
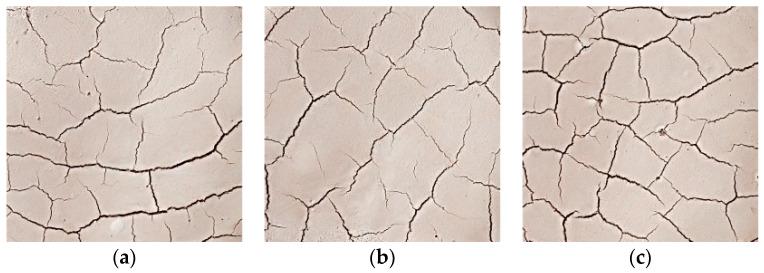
Improved soil fissure development morphology. (**a**) Group S10 specimens; (**b**) Group S11 specimens; (**c**) Group S12 specimens.

**Figure 9:**
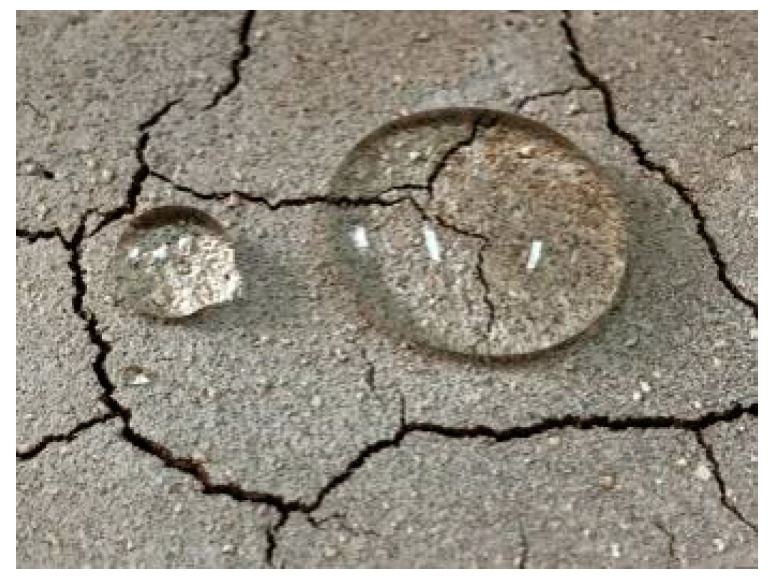
Soil sample Hydrophobic rendering.

Figure 8 shows the morphology of fissures in improved soil samples S10–S12. As can be seen from the fissure morphologies of S1–S3 soil samples in Figure 4, the number of dry shrinkage fissures in the improved soil is smaller and the width is smaller. Figure 9 shows the hydrophobic effect of the modified soil samples. It can be seen from Figure 9 that, when dripping water on the surface of the modified soil sample after drying and shrinking, the water can maintain the shape of the water droplet and not penetrate into the soil. It can be seen that the modified soil also has good hydrophobicity.

## 3. Results

### 3.1. Effect of Particle Size on Dry Shrinkage Fissures

**Figure 10:**
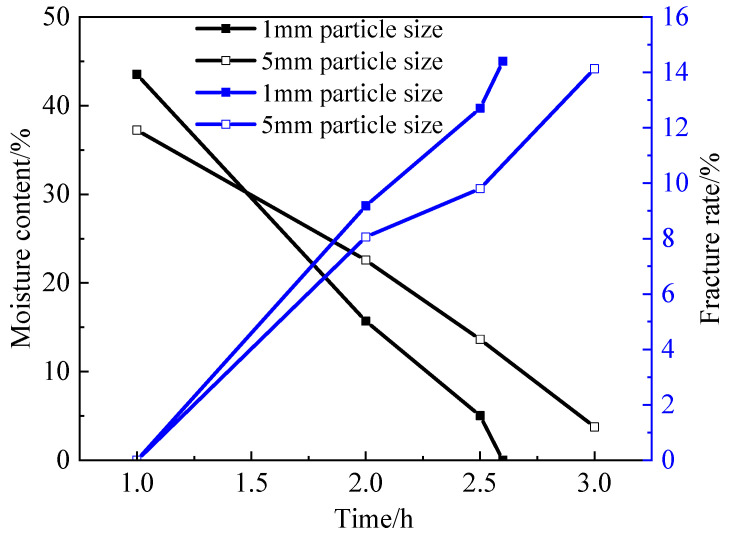
The relationship curves of fissure rate, moisture content and time.

The effect of particle size on drying shrinkage fissures was studied by the quantitative analysis of the surface fissure ratio of the soil samples. Both the moisture content and the surface fissure rate are parallel averages. Table 2 and Table 3 are the fissure rates of soil samples S1–S6, respectively, where the time in the table is the duration of the test. Figure 8 shows the relationship curves of fissure rate, moisture content and time. From Table 2, Table 3, and Figure 10, it can be seen that the evaporation rate and surface fissure rate of the moisture increase with the increase in the soil particle size. Soil samples with a grain size of 1 mm (groups S1–S3) cracked more severely than those with a grain size of 5 mm (groups S4–S6), especially at the late stage of cracking when h = 2.5, the cracking rate of soil samples from groups S1–S3 was 2.91% higher than that of soil samples from groups S4–S6, and the water content of soil samples from groups S1–S3 was 8.59% lower than that of soil samples from groups S4–S6. water content was 8.59% lower than that of the soil samples in groups S4–S6. Both of these values are maximum values throughout the experiment, proving that the larger the soil sample particles, the more likely the soil sample will lose water and will be the first to experience cracking.

### 3.2. Effect of Dry and Wet Cycle on Dry Shrinkage Fissures

**Figure 11:**
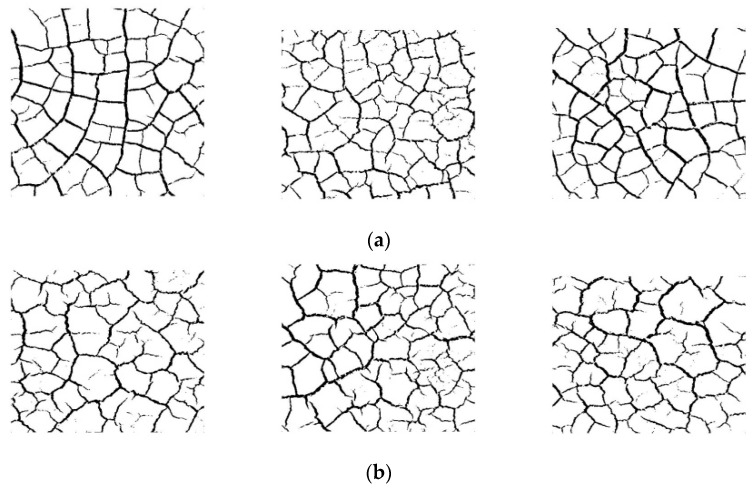

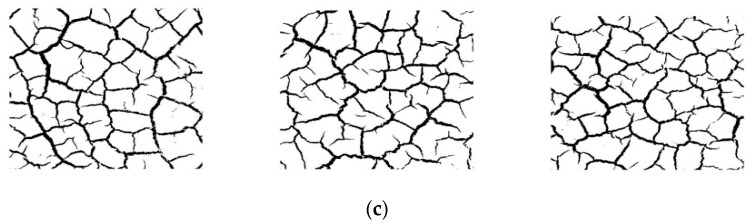
Soil sample wet and dry cycle binarized fissure diagram. (**a**) The fissure morphology of the first dry–wet cycle of soil samples; (**b**) the fissure morphology of the second dry–wet cycle of soil samples; (**c**) the fissure morphology of the third dry–wet cycle of soil samples.

Figure 11 shows the fissure morphology of soil samples S1–S3 under three dry–wet cycles. As can be seen from Figure 11, with the increase in the number of dry–wet cycles, the number of fissures gradually increases and the width of the fissures increases.

### 3.3. Effect of Thickness on Dry Shrinkage Fissures

**Figure 12:**
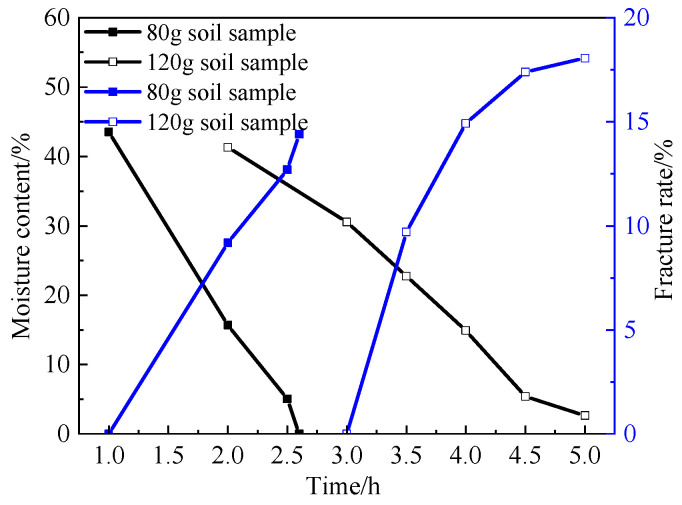
Comparison curves of moisture content and fissure rate of soil samples of two thicknesses.

Table 4 shows the relationship between dry shrinkage crack rate, water content and time of the 3 cm soil samples. It can be seen from Table 4 and Figure 12 that the samples with thicker soil layers (S7–S9) did not crack until 3 h, while the samples with thinner soil layers (S1–S3) did not crack until 1 h after the test. This shows that the thicker the soil sample is, the longer the time required for complete water evaporation. In addition, the crack rate of the samples with thicker soil layers (S7–S9) increased by 18.07% within two hours after cracking, which is 2.95% higher than that of the samples with thinner soil layers (S7–S9). This shows that the thicker the soil sample is, the greater the surface crack rate is.

### 3.4. Dry Shrinkage Fissure Test of Improved Soil

**Figure 13:**
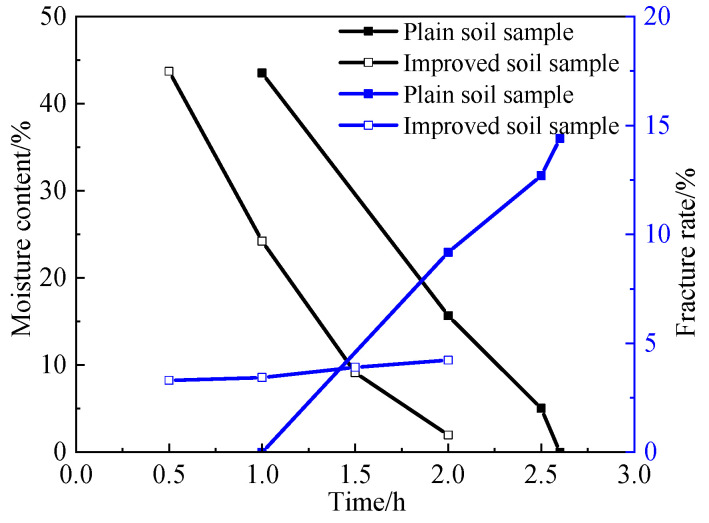
Comparison curve between the improved soil and the plain soil.

The earthen soil was improved by lime and silicone water repellents, which not only reduced the fissure rate but also increased the waterproofness of the soil sample, and the surface of the soil sample showed a good hydrophobic effect. Table 5 shows the relationship between moisture content, fissure rate and time of the improved soil. Figure 13 is the comparison curve between the improved soil and the plain soil. As can be seen from Table 5 and Figure 13, the surface fissure rate of the improved soil samples decreased, indicating that the lime and silicone water repellents can effectively inhibit the drying shrinkage and cracking of the soil.

## 4. Discussion

The formation and development of fissures is a major factor in the structural damage of soils. Currently, research on the cracking and improvement of site soils focuses only on improving strength and mechanical properties, while ignoring the durability of the soil site proper. Cui and Chen et al. [20,21,22], experts in the field of heritage conservation, have reinforced earthen sites by using nontoxic and harmless materials such as quicklime, a modified polyvinyl alcohol (SH) consolidant, and potassium silicate (PS) materials, with obvious reinforcement effects. However, all of the above mentioned authors used drilling and grouting to inject reinforcement materials, which can cause secondary damage to the body of the earthen site and is not conducive to the conservation and repair of cultural relics. In this paper, based on image processing techniques [23], we investigated the cracking pattern on the surface of earthen sites [24,25] and selectively sprayed reinforcement materials on the surface. This approach can be more efficient in finding the most severely cracked areas and fissure development paths [26], with targeted reinforcement measures.

The Cai Guo Forbidden City earthen site is located in the Central Plains region of China, where it is rainy in summer and cold in winter. In addition, the site soils are expansive soils and the water resistance of the earthen site body needs to be considered [27] to improve the durability of the earthen site. In order to allow the reinforcement of different parts of the earthen site, dry shrinkage cracking tests were carried out, in this paper, for different soil thicknesses and soil sample particles to investigate the crack development pattern of the earthen site under different working conditions and to provide experimental support for subsequent reinforcement.

## 5. Conclusions

Through the dry shrinkage test of expansive soil in the ruins of the ancient city of Caiguo, the effects of particle size, thickness, and number of dry–wet cycles on the dry-shrinkage fissures of soil samples were studied. Lime and silicone hydrophobic agents effectively reduce the generation of dry shrinkage fissures in soil samples, and have a good waterproof effect. We obtained the following results:

(1) The particle size of soil sample has a direct impact on the crack rate of expansive soil. In the process of drying, the larger the particle size of a soil sample is, the more cracks will be produced after drying shrinkage. Especially in the late stage of cracking, the cracking rate of soil samples from groups S1–S3 was 2.91% higher than that from groups S4–S6, and the water content of soil samples from groups S1–S3 was 8.59% lower than that from groups S4–S6. Both of these values are maximum values throughout the experiment, proving that the larger the soil sample particles, the more likely the soil sample will lose water and will be the first to experience cracking.
(2) A comparison of 1, 2 and 3 dry and wet cycles of cracks shows that as the number of dry and wet cycles increases, the distance between the main cracks increases, the number of cracks from the cracks increases significantly and also develops gradually, and the distribution of cracks becomes more and more uniform.
(3) Thickness has a significant effect on the moisture content, crack width and surface crack rate of the samples. The thicker the thickness is, the wider the crack width is, and there are many warps of different sizes on the edge of soil particles on the surface of soil sample. The soil samples with the low soil layer thickness (S1–S3 groups) will crack earlier than the soil samples with the large soil layer thickness (S4–S6 groups), but the crack rate of the soil samples with the large soil layer thickness is 2.95% higher than that of the soil samples with the small soil layer thickness. This shows that the thicker the soil sample is, the longer the time required for complete water evaporation and the greater the surface crack rate.
(4) The improvement in the mixed admixture produces crystals between the soil particles, increases the compactness, improves the strength, effectively inhibits the generation of cracks, and makes the soil sample have good hydrophobicity, which weakens the adverse effect of water on the soil.

## Figures and Tables

**Table 1:** Basic physical indicators of soil samples.

Liquid Limit/%	Plastic Limit/%	Plasticity Index	Free Expansion Rate/%	Natural Moisture Content/%
34.7	15.2	19.5	58	23.5

**Table 2:** The relationship between the time and fissure rate of S1, S2 and S3 of soil samples with particle size of 1mm.

	Number	S1	S2	S3	Average Value
Time (h)					
1		41.91% (0%)	44.41% (0%)	44.24% (0%)	43.52% (0%)
2		15.10 (10.21%)	16.19% (8.07%)	15.82% (9.29%)	15.70% (9.19%)
2.5		3.77% (14.32%)	4.90% (11.46%)	6.49% (12.36%)	5.05% (12.71%)
2.6		0% (15.31%)	0% (13.38%)	0% (14.53%)	0% (14.41%)

**Table 3:** The relationship between the time and fissure rate of S4–S6 of soil samples with particle size of 5 mm.

	Number	S4	S5	S6	Average Value
Time (h)					
1		38.71% (0%)	37.72% (0%)	35.29% (0%)	37.24% (0%)
2		24.25% (8.11%)	23.48% (8.58%)	19.99% (7.49%)	22.57% (8.06%)
2.5		15.41% (9.55%)	15.35% (9.66%)	10.16% (10.18%)	13.64% (9.80%)
3		5.53% (13.81%)	2.87% (13.81%)	2.91% (14.77%)	3.77% (14.13%)

**Table 4:** The relationship between the dry shrinkage fissure rate, moisture content and time of the soil samples.

	Number	S7	S8	S9	Average Value
Time (h)					
2		42.17% (0)	41.26% (0%)	40.56% (0%)	41.33% (0%)
3		31.52% (0)	29.99% (0%)	30.55% (0%)	30.55% (0%)
3.5		22.49% (7.45%)	22.58% (12.79%)	22.77% (8.90%)	22.77% (9.8%)
4		14.24% (13.72%)	14.46% (18.27%)	14.92% (12.76%)	14.92% (14.92%)
4.5		4.99% (16.11%)	5.31% (19.83%)	5.43% (16.28%)	5.43% (17.40%)
5		2.47% (17.26%)	2.60% (20.17%)	2.68% (16.77%)	2.68% (18.07%)

**Table 5:** Relationship between moisture content, fissure rate and time of the improved soil.

	Number	S10	S11	S12	Average Value
Time (h)					
0.5		45.84% (2. 61%)	41.54% (3.31%)	43.81% (4.02%)	43.73% (3.31%)
1		26.71% (2.74%)	21.45% (3.38%)	24.60% (4.19%)	24.25% (3.44%)
1.5		11.62% (3.09%)	7.23% (3.77%)	8.59% (4.86%)	9.15% (3.91%)
2		3.41% (3.47%)	0.65% (4.10%)	1.92% (5.16%)	1.99% (4.24%)

## Data Availability

The data presented in this study are openly available within the document.
